# Bloodmeal Analysis Reveals Avian *Plasmodium* Infections and Broad Host Preferences of *Culicoides* (Diptera: Ceratopogonidae) Vectors

**DOI:** 10.1371/journal.pone.0031098

**Published:** 2012-02-20

**Authors:** Diego Santiago-Alarcon, Peter Havelka, Hinrich Martin Schaefer, Gernot Segelbacher

**Affiliations:** 1 Biología y Conservación de Vertebrados, Instituto de Ecología A.C., Xalapa, Veracruz, México; 2 Department of Zoology, Staatliches Museum für Naturkunde, Karlsruhe, Baden-Württemberg, Germany; 3 Department of Ecology and Evolutionary Biology, University of Freiburg, Freiburg, Baden-Württemberg, Germany; 4 Department of Wildlife Ecology and Management, University of Freiburg, Freiburg, Baden-Württemberg, Germany; Institut Pasteur, France

## Abstract

Changing environmental conditions and human encroachment on natural habitats bring human populations closer to novel sources of parasites, which might then develop into new emerging diseases. Diseases transmitted by host generalist vectors are of special interest due to their capacity to move pathogens into novel hosts. We hypothesize that humans using forests for recreation are exposed to a broad range of parasites from wild animals and their vectors. A corollary of this is that new vector-host, parasite-host, and vector-parasite associations could eventually develop. Thus, we expect to observe atypical vector-host associations. Using molecular bloodmeal analysis via amplification of the mtDNA COI gene we identified the vertebrate hosts of *Culicoides* (Diptera: Ceratopogonidae) species in a sub-urban forest of Southwestern Germany. Bloodmeals were also checked for haemosporidian infections by amplifying a fragment of the mtDNA cyt *b* gene. We identified a total of 20 *Culicoides* species, thirteen of which fed on humans. From 105 screened bloodmeals we obtained high quality sequences for 77 samples, 73 (94.8%) originated from humans, two from livestock (*Bos taurus* and *Equus caballus*), and two from wild birds (*Sylvia atricapilla* and *Turdus merula*). We found that four *Culicoides* species previously assumed to feed exclusively on either birds (*C. kibunensis*) or domestic mammals (*C. chiopterus*, *C. deltus*, *C. scoticus*) fed also on humans. A total of six *Culicoides* abdomens were infected with avian haemosporidian parasites (*Plasmodium* or *Haemoproteus*), four of those abdomens contained blood derived from humans. Our results suggest that parasites of wild animals may be transferred to humans through infectious bites of *Culicoides* vectors. Further, we show that *Culicoides* vectors believed to be a specialist on specific vertebrate groups can have plastic feeding preferences, and that *Culicoides* are susceptible to infection by *Plasmodium* parasites, though vector viability must still be experimentally demonstrated.

## Introduction

Haemosporidian parasites are responsible for millions of infections and thousands of deaths in humans, as well as domestic and wild animals each year [1], [2]; this is a pattern that is becoming more relevant on the face of environmental changes because warming climate is helping the geographic expansion of both parasites and vectors into regions where they were previously absent [3]. Despite its relevance for human and animal health, knowledge on the insect vectors feeding on different vertebrate species and transmitting haemosporidians is poor [1]. The lack of knowledge on insect vectors transmitting blood parasites creates two challenges: First, as the insect vectors of most of the haemosporidian parasites described to date are unknown [1], we do not know how and with whom these vectors are directly and indirectly interacting with. Second, if vectors sucking blood from humans also feed on other vertebrates (e.g., [4]–[6]), humans will be exposed to a wide range of parasites existing in the animal community surrounding them (e.g., [7]). Furthermore, host specificity of a parasite can be altered by selection [8]–[10] (e.g., it is possible to experimentally adapt an avian parasite (*Plasmodium lophurae*) to a mammal host [11]), which means that parasites are capable to adapt naturally to new hosts, including humans. It is important to know what blood parasites make it into the blood stream of humans, even if initially they do not develop into an infection, because this is the first step towards new emerging diseases (e.g., West Nile Virus [12],). However, our current knowledge on what vectors are feeding on humans is insufficient, and thus we cannot assess which parasites and their associated diseases represent a possible threat to human health.

Studies on host feeding preferences have demonstrated that many species of *Culicoides* (i.e. biting midges) are specialized on either birds (e.g. *C. kibunensis*) or mammals (e.g. *C. chiopterus*, *C. deltus*), but there are also some species that are host generalists (e.g. *C. festivipennis*, *C. obsoletus*) [13]–[19]. Host generalist biting midges are of special interest because they are capable of feeding on different vertebrate groups, and thus, can facilitate the emergence of new diseases. Furthermore, under unfavorable or altered ecological conditions even vectors specialized on a specific vertebrate group can be found to feed on suboptimal hosts [6]. Here, we studied feeding preferences of *Culicoides* (Diptera: Ceratopogonidae) vectors in a sub-urban forest (Mooswald) of the city of Freiburg in Southwestern Germany. This forest is embedded in a matrix of buildings and farms, and humans frequently use it for recreational purposes.

Human encroachment on natural habitats provides opportunities for novel pathogens to “jump” into human populations. We hypothesize that humans using sub-urban forests are exposed to a broad range of wild animal parasites and their vectors. If many vectors are feeding on different hosts (i.e. generalists) we expect to observe hitherto unknown vector-host associations under these conditions (e.g. bird sucking *Culicoides* feeding on humans). A corollary of this is that new vector-host, host-parasite, and vector-parasite associations could develop even if vectors cannot currently transmit novel parasites (i.e. not competent vectors). This would represent the first step towards new ecological interactions. In our study we address the following questions: 1) what species of *Culicoides* vectors are present in the study area? 2) On what vertebrate species are *Culicoides* vectors feeding on? And 3) what haemosporidian parasites are infecting *Culicoides* vectors?

## Results

We identified 20 *Culicoides* species in the Mooswald forest in a total of 853 *Culicoides* specimens. We had a total of 105 engorged (i.e. insects with a full or partial bloodmeal) *Culicoides* midges from 13 different species (Table 1). We were able to obtain good quality COI sequences from 77 bloodmeals; most likely the DNA in the other 28 bloodmeals was degraded due to an advanced digestion stage in the vector's mid gut. Seventy-three blood meals were derived from humans, two from birds (*Sylvia atricapilla* and *Turdus merula*), and two from livestock (cow and horse). Six of these 77 abdomens with bloodmeals were infected with avian haemosporidian parasites (Table 2). Four haemosporidian-infected abdomens contained blood derived from humans, of which two were infected with avian *Plasmodium* [100% similar to lineage LINN1 and 99% similar to lineage WA38, see also Table 2] and two with *Haemoproteus* [99% similar to *Haemoproteus* sp. lineage CCF2 from *Fringilla coelebs* and 100% similar to *Haemoproteus parabelopolskyi* haplotype SYAT02 from *Sylvia atricapilla*]. The other two infected abdomens were assigned as bloodmeals from birds; *Turdus merula* was infected with a lineage 99% similar to *H. minutus* and *H. pallidus* and *Sylvia atricapilla* was infected with lineage SYAT07 (most likely *H. parabelopolskyi*).

**Table 1:** Bloodmeals from *Culicoides* vector species found in the Mooswald forest of Freiburg, Germany.

Vector species	Number of bloodmeals*	Vertebrate host detected in bloodmeals**	Previously known vertebrate hosts***
***Culicoides clastrieri***	5	*Homo sapiens* (3)	NI
***C. chiopterus***	2	*Homo sapiens* (1)	Cows [14], [15], [17], [19], [39]
***C. deltus***	2	*Homo sapiens* (1)	Horses and Cows [19]
***C. dewulfi***	5	*Homo sapiens* (2)	NI
***C. duddingstoni***	0		NI
***C. festivipennis***	6	*Homo sapiens* (3)	Cows, Man, Birds [17], [19], [40], [41]
***C. heliophilus***	0		Man, Cows, Sheep, Dogs [13]–[15], [19], [34]
***C. kibunensis***	8	*Sylvia atricapilla* (1)*Homo sapiens* (7)	Birds [16], [17], [19]
***C. newsteadi***	0		Man, Poultry [19], [42]
***C. obsoletus***	24	*Bos Taurus* (1)*Homo sapiens* (16)	Birds, Man, Livestock [13], [16], [18], [19], [39], [43]
***C. pallidicornis***	1	*Homo sapiens* (1)	Sheep, Cows, Birds, Man [13], [14], [19], [21], [43], [44]
***C. pictipennis***	23	*Homo sapiens* (14)*Turdus merula* (1)	Birds, Man [13], [16], [17], [19], [43]
***C. poperinghensis***	8	*Homo sapiens* (7)	NI
***C. pseudoheliophilus***	0		NI
***C. pulicaris***	4	*Homo sapiens* (3)	Cows, Sheep, Horses, Buffaloes, Man [13]–[15], [19], [34], [39], [43]
***C. punctatus***	0		Man, Cows [17], [19], [39]
***C. scoticus***	9	*Equus caballus* (1)*Homo sapiens* (7)	Cows, Horses, Sheep [19]
***C. semimaculatus***	1	*Homo sapiens* (1)	NI
***C. stigma***	0		Cows, Man [19], [21], [39]
***C. vexans***	0		Horses, Goats, Man, Birds [14], 15,19,44

NI  =  no information available.

*We were unable to determine the vector species for 7 blood meals; all seven contained human blood.

**Number of times a vertebrate host was identified from a bloodmeal is in parenthesis.

***Reference information for previously reported hosts is provided as a superscript.

**Table 2:** Haemosporidian parasites found in bloodmeals from *Culicoides* species collected in the Mooswald forest of Freiburg.

Vector Species	Vertebrate host identified from haemsoporidian-infected bloodmeal	Haemosporidian parasite lineage*
***C. kibunensis***	*Sylvia atricapilla*	100% similar to *Haemoproteus* sp. haplotype SYAT07 from *Sylvia atricapilla* (AY831754)
***C. pictipennis***	*Homo sapiens*	100% similar to avian *Plasmodium* haplotype LINN1 (GQ471953)
***C. pictipennis***	*Turdus merula*	99% similar to *Haemoproteus minutus* (DQ630013) and *Haemoproteus pallidus* (DQ630005)
***C. poperinghensis***	*Homo sapiens*	100% similar to *Haemoproteus parabelopolskyi* haplotype SYAT02 from *Sylvia atricapilla* (AY831751)
***C. semimaculatus***	*Homo sapiens*	99% similar to *Haemoproteus* sp. haplotype CCF2 from *Fringilla coelebs* (AF495551)

Haemosporidian sequences GenBank™ accession numbers are in parenthesis.

*We were not able to identify the vector species for one of the infected bloodmeals, this bloodmeal contained human blood and a haemosporidian sequence 99% similar to several avian *Plasmodium* haplotypes (EU810633, DQ847271, HQ453998, HQ454001, HQ454003, AB477124, JF411406).

In the case of vectors, one abdomen from *C. pictipennis* was infected with avian *Plasmodium* lineage LINN1, and the other *Plasmodium* infected abdomen was obtained from an unidentified *Culicoides* specimen (Table 2). We identified 13 *Culicoides* species with bloodmeals derived from humans. We found a *Culicoides* species previously known to feed only on birds (*C. kibunensis*) with 7 human bloodmeals, 3 *Culicoides* species reported to feed only on domestic (livestock and pets) mammals (*C. chiopterus*, *C. deltus*, *C. scoticus*) with 9 human bloodmeals, and 3 host generalists (for birds and mammals) *Culicoides* species (*C. festivipennis*, *C. obsoletus*, *C. pallidicornis*) with 20 human bloodmeals (Table 1).

## Discussion

Our results show two important findings. First, *Culicoides* vectors have broad host feeding preferences, even for those species that have been assumed to feed exclusively on birds or mammals, which opens the possibility for transferring parasites across distantly related vertebrate hosts, including humans. It has been suggested that some vectors from different Diptera families have restricted host feeding preferences (e.g. ornithophilic), but we suggest that specificity is less than previously assumed and may partly reflect a biased research agenda towards a limited set of vectors and hosts (e.g., [1], [2]), and that many vectors around the world probably will have to adapt to new hosts in the light of rapid environmental changes because depending on their ecology they will experience a geographical range expansion or contraction. For example, *C. kibunensis* is known to be an ornithophilic vector, but here we show that seven *C. kibunensis* individuals sucked blood from humans; given that humans are a common source of blood no matter the surrounding conditions, it is likely that this vector species will be resilient to abrupt environmental changes. Laboratory experiments have already demonstrated that many vectors with specific vertebrate host feeding preferences can successfully feed on alternative, probably sub-optimal, vertebrate hosts and transfer parasites across distantly related vertebrates [4], [5], [9]–[11]. Some vector species obviously have plastic feeding preferences, which are adjusted depending on the availability of host species [6]. Here, we have shown that some *Culicoides* species under natural conditions are readily feeding on humans (Table 1), which presumably is a suboptimal host based on previous knowledge (see references from Table 1). Thus, our results provide a first important link from previous experimental findings in the laboratory [4], [5], [9]–[11] to changing host parasite interactions in the wild.

In areas with some degree of modification (e.g. sub-urban forests) infectious vectors can bite novel hosts that can turn to be suboptimal for both the parasite and the vector (i.e. host blood factors can be detrimental for vectors). In the case of sub-urban forests or other areas with high densities and abundance of humans, humans can serve as a blood source that is a bigger and more readily available target than the normal wild animal source. Thus, humans can intercept vector infectious bites and can be acting either as a dead-end or as a highly mobile reservoir capable of carrying viable wild animal parasites to other regions. Although *Culicoides* vectors infected with avian haemosporidians could transmit these parasites to humans, it is difficult to predict if a disease will eventually develop because parasites not adapted to the immune system of novel hosts can be rapidly detected and cleared [20].

Thus, we argue that some future studies should focus on the transmission of putative non-human parasites into humans and should identify the viability of such blood parasites within the new host system.

Second, results showed that avian haemosporidian parasites (*Plasmodium* sp. and *Haemoproteus* sp.) were infecting abdomens of *Culicoides* vectors, four of which contained blood derived from humans. Our observations would suggest two alternative non-exclusive explanations, first, that a vector infected with avian haemosporidians can take a blood meal from a human and inject the sporozoites into the human host, which subsequently can remain in circulation and be swallowed by another vector. Previous attempts to infect humans with avian *P. relictum* and monkey *P. cynomolgi* in the laboratory were unsuccessful [5], [21], but parasites remained alive in the peripheral human blood and were infectious to pigeons up to 4.5 hours after inoculation [21]. Sporozoites injected by vectors into the peripheral blood of vertebrate animals can remain in circulation for days [22]; therefore, sensitive PCR methods can be able to amplify the DNA of a few circulating sporozoites that might eventually not develop into an infection in the vertebrate host [22], but that still can cause an immunological reaction. Thus, the avian haemosporidian DNA detected in abdomens containing human blood might have originated from circulating sporozoites, and in the case of *Plasmodium* parasites by merogonic (i.e. merozoites) asexual stages as well, swallowed by the vectors instead of gametocytes. Second, the avian haemosporidian DNA detected in vector abdomens that contained human blood might have derived from vector's mid gut oocysts. This is a likely possibility since we used entire insect abdomens to extract the DNA from the bloodmeal; it was impossible to separate the tiny bloodmeal from the abdomen of midges preserved in alcohol, which can be more easily done using fresh insect material. Although we cannot clearly differentiate between the two different explanations it is important to note that vectors carrying avian haemosporidians are readily sucking blood from humans. Furthermore, experimental studies have demonstrated that an avian parasite, *P. lophurae*, can adapt and be viable in mice just after four rounds of infectious inoculations [11], which suggests that haemosporidian parasites can adapt to new distant hosts rather quickly. In particular, this is important because the potential of parasites to rapidly adapt to new hosts will be one of the greatest challenges to human and animal health, as well as of increasing importance in the study of zoonotic diseases. Hence, it is a priority to study the biodiversity of haemosporidian parasites in wild vertebrates and of the vectors transmitting them since we must first know what is out there in order to be prepared for any epidemiological emergency.

*Culicoides* vectors transmitting bluetongue virus (BTV, Reoviridae: *Orbivirus*) were detected in Central Europe in 2006 [23]. This is a disease of domestic livestock and wild ruminants that can create substantial economic problems [23], [24]. *C. obsoletus*, *C. pulicaris*, and *C. scoticus* are considered the main vectors of BTV in Germany and their bloodmeals derived only from livestock [24], [25]. In our study we found *C. obsoletus* to be the vector with the highest number of bloodmeals, and together with *C. scoticus* and *C. pulicaris* fed mainly on humans (Table 1). Given that BTV is already established in Germany, BTV could potentially also invade human blood via infectious *Culicoides* bites, though BTV apparently does not produce acute illness in humans [26].

The genus *Orbivirus* (Arbovirus: Reoviridae) is comprised by viruses that are highly pathogenic to wild and domestic ruminants (e.g. African horse sickness virus (AHSV), equine encephalosis virus (EEV)), but that can generate only mild fevers in humans or subclinical cases, and some can create acute diarrhoeal illness in man [27]. Despite BTV being apparently mild to humans, this example serves to illustrate the potential problems that a more aggressive disease can cause. For example, other Arbovirus families can create more severe diseases in man such as St.Louis encephalitis virus (Arbovirus: Flaviviridae) and La Crosse encephalitis virus (Arbovirus: Bunyaviridae) [28].

Currently, it is widely accepted that vectors from specific Diptera families transmit haemosporidians only from specific genera [1], [29]. For example, avian haemosporidians from the genus *Haemoproteus* and *Leucocytozoon* (sub-genus *Akiba*) are considered to be transmitted only by *Culicoides* vectors [1], [29]. We have shown here that *Culicoides* vectors can carry *Plasmodium* parasite DNA. Although our data do not prove that *Culicoides* vectors are able to transmit avian *Plasmodium* parasites, other researchers also found *Culicoides* species infected with *Plasmodium* parasites, and in their case infection was detected in the head-thorax portion, indicating most likely that *Plasmodium* sporozoites were present in the salivary glands of those *Culicoides* vectors [30]. Moreover, mosquitoes (Culicidae) are considered to transmit *Plasmodium* parasites only, but *Haemoproteus* parasite DNA has been detected in mosquitoes from different genera (*Anopheles*, *Aedes*, *Verrallina*, *Culex*, *Coquillettidia*) [31], [32]. Together, these studies suggest that our current knowledge about vector-family specificity on distinct haemosporidian genera (e.g. *Plasmodium*-Culicidae, *Haemoproteus* (*Parahaemoproteus*)-Ceratopogonidae) might need to be revised. To verify vector competence further experimental infections on natural vector-parasite-host associations are needed.

In conclusion, we have documented that *Culicoides* vectors infected with avian haemosporidians readily feed on humans. *Culicoides* have plastic feeding preferences and can adapt to feed on many different animals, even across vertebrate groups. Additionally, previous studies [30] have already demonstrated that *Culicoides* vectors can be infected by *Plasmodium* parasites. However, it is as yet unclear how widespread that phenomenon is and further experimental studies are needed to investigate if *Plasmodium* parasites are truly transmitted by *Culicoides* species. Our results highlight the need for basic biodiversity and experimental studies of haemosporidian parasite systems, and give way to the urgency of parasitological studies under the current framework of rapid environmental change. For humans, this would represent the establishment of monitoring programs using serological tests to pick up exposure to wild animal parasites.

## Materials and Methods

We collected *Culicoides* midges during 2010 using BG Sentinel® traps (Regensburg, Germany) with UV light. Collections were made in a sub-urban forest of Freiburg in Southwestern Germany (Mooswald Forest, 48° 00 N, 07° 51 E). This forest is a mixed deciduous forest with water available in small streams and ponds where Diptera vectors can develop. The forest contains many walking trails including some forest roads. Traps were operated overnight starting at 18:00 hrs, and picked up next morning at 08:00 hrs. We sampled from early April until the end of September conducting a total of 32 trapping nights using two traps, which yielded 64 samples. Samples were subsequently processed in the lab with a stereomicroscope; each *Culicoides* sample was divided into engorged (i.e. insects with a full or partial bloodmeal) and unfed midges. Engorged midges were used for bloodmeal analysis. We dissected the abdomen of each individual midge; dissecting tools were sterilized with alcohol and fire for every insect. We extracted the DNA from the blood contained in the abdomen and conducted PCR (see below). The possibility exists that midges that previously had a bloodmeal (i.e. parous) were infected with haemosporidian parasites, in which case different developmental stages can be present in the insects mid gut's wall (i.e. oocysts) or in the salivary glands (i.e. sporozoites), which would imply that these vectors could inject haemosporidian sporozoites during subsequent bloodmeals. The thorax, head, and genitals of dissected midges were saved in 70% alcohol for subsequent morphological identification. Unfed midges were kept in 70% alcohol until they were processed for identification. Determination was conducted using taxonomic keys [14], [15], [33]–[35]. Representative *Culicoides* specimens were mounted on slides and they are kept in the insect collection of Havelka and Aguilar at the Staatliches Museum für Naturkunde in Karlsruhe, Germany.

We extracted the DNA from the bloodmeals of the *Culicoides* midges using the DNeasy Blood and Tissue® kit (QIAGEN, Hilden). We conducted a nested PCR to amplify the bar code (COI mitochondrial DNA gene) of each bloodmeal using the protocol described in [36], which amplifies ∼758-bp. We also used parasite genus-specific primers in a nested PCR protocol that amplifies a fragment of ∼524-bp of the cytochrome *b* mitochondrial DNA gene of haemosporidian parasites to verify if vector abdomens were infected [37]. All PCRs were run with a positive (blackcap haemosporidian infected blood sample) and a negative control (ddH_2_O). Subsequently, we cleaned the PCR products with the MinElute kit (QUIAGEN, Hilden), placed them in a 96-well plate and sent out for sequencing at Microsynth AG, Switzerland. Sequences were edited with 4Peaks v.1.7.2 (mekentosj.com). We used the identification engine of the BOLD (Barcode of Life Data Systems) systems v2.5 [38] to identify the vertebrate species for each COI sequence obtained from the bloodmeals. Cyt *b* sequences obtained from haemosporidian-infected bloodmeals were compared against DNA sequences available in GenBank™ by using the BLAST algorithm of the NCBI (National Center for Biotechnology Information) database.
